# Action Video Game Playing Is Reflected In Enhanced Visuomotor Performance and Increased Corticospinal Excitability

**DOI:** 10.1371/journal.pone.0169013

**Published:** 2016-12-22

**Authors:** Olivier Morin-Moncet, Jean-Marc Therrien-Blanchet, Marie C. Ferland, Hugo Théoret, Greg L. West

**Affiliations:** 1 Department of Psychology, Université de Montréal, Montréal, Canada; 2 Hôpital Sainte-Justine Research Center, Montréal, Canada; University of Bologna, ITALY

## Abstract

Action video game playing is associated with improved visuomotor performance; however, the underlying neural mechanisms associated with this increased performance are not well understood. Using the Serial Reaction Time Task in conjunction with Transcranial Magnetic Stimulation, we investigated if improved visuomotor performance displayed in action video game players (actionVGPs) was associated with increased corticospinal plasticity in primary motor cortex (M1) compared to non-video game players (nonVGPs). Further, we assessed if actionVGPs and nonVGPs displayed differences in procedural motor learning as measured by the SRTT. We found that at the behavioral level, both the actionVGPs and nonVGPs showed evidence of procedural learning with no significant difference between groups. However, the actionVGPs displayed higher visuomotor performance as evidenced by faster reaction times in the SRTT. This observed enhancement in visuomotor performance amongst actionVGPs was associated with increased corticospinal plasticity in M1, as measured by corticospinal excitability changes pre- and post- SRTT and corticospinal excitability at rest before motor practice. Our results show that aVGPs, who are known to have better performance on visual and motor tasks, also display increased corticospinal excitability after completing a novel visuomotor task.

## 1. Introduction

Video games have become an increasingly popular activity, with individuals now spending an average of 3 billion hours per week of game playing worldwide [1]. Because of this, there is a growing interest in understanding how the exposure to video games affects cognitive processes and their underlying neural mechanisms [2–9]. Specifically, action video game (actionVG) playing has been shown to increase performance in cognitive tasks within several domains including visual attention [2,9,10], visual short-term memory [11], executive function [5] and procedural learning abilities [12,13]. At this point, little is known about the neurophysiological mechanisms underlying observed behavioral differences in performance between action video game players (actionVGPs) and non video game players (nonVGPs). One hypothesis is that enhanced visuomotor performance in actionVGPs might involve the modulation of underlying neuroplasticity mechanisms through repeated practice at a visuomotor task [13]. This could be particularly true in the visuomotor domain as actionVGPs have been shown to have enhanced fine motor skills [14], oculomotor accuracy [7] and reaction times when detecting visual stimuli [2,10,15,16].

To date, no physiological evidence has linked the higher visuomotor performance observed in actionVGPs to enhanced neuroplasticity. One way to study the changes in neuroplasticity that are associated with motor practice is to measure corticospinal excitability with transcranial magnetic stimulation (TMS). Applied over the primary motor cortex (M1), focal single-pulse TMS allows measurement of corticospinal changes in excitability by measuring motor-evoked potentials (MEP) with electrodes placed on the contralateral muscle [17–19]. Furthermore, the Serial Reaction Time Task (SRTT) is widely used to assess both overall visuomotor performance and procedural motor learning [20–24]. The M1 region contralateral to the active hand is particularly active during the SRTT [20–24]. Incidentally, TMS studies have documented increases in MEP amplitudes resulting from the stimulation of the first dorsal interosseous (FDI) muscle’s cortical reprensentation in M1 after completing the SRTT [22–24]. This reflects increased cortical excitability in M1, which occurs as a person practices the SRTT, and is associated with cortical plasticity that is hypothesized to mediate enhanced visuomotor performance [25–28].

Previous research has shown that actionVGPs display better visual attention and motor control performance [2,9,10,12,13]. In the current study, we therefore tested if actionVGPs would also show increased corticospinal plasticity in M1 after completing a novel visuomotor task, namely the SRTT paradigm [22,24]. We predicted that after completing the SRTT with their right hand, both actionVGPs and nonVGPs should display increased corticospinal excitability over the left cortical representation of the right FDI muscle in M1. However, we predicted a greater increase in the left M1 corticospinal excitability among actionVGPs compared to the nonVGP group.

## 2. Materials and Methods

### 2.1. Participants

Twenty-four individuals (9 female) participated in the study. As described in West et al. (2015), an extensive phone questionnaire that included various components such as demographic information, vision, medical history, cardiovascular diseases, neurological disorders, medical conditions, psychiatric disorders, substance abuse, general medication, family history, and handedness was administered to potential participants. Participants were excluded from the study if they had a history of neurological or psychiatric disorders including depression and anxiety. Participants were also excluded if they had a history of substance abuse (recreational drugs, alcohol consumption that exceeds 10 alcoholic beverages per week, and cigarette use that exceeds 10 cigarettes per day) or a history of medical conditions that includes hormone disorders, cancer, cardiovascular disease, diabetes and presented no contraindication to the safe use of TMS [29]. All participants gave written informed consent to undergo the experimental procedures, which were approved by ethics board at the University of Montreal. All participants were non-musicians due to previous evidence that musicianship can influence corticospatial plasticity during the SRTT task.

Participants were placed into the actionVGP or nonVGP group on the basis of a questionnaire about their video game playing habits. To be considered an actionVGP, a participant needed to report a minimum of 6 hours a week of *action* video game playing during the previous 6 months [2–4,6–9,12,30,31]. An abridged list of actionVGs participants reported playing includes first-person shooters such as Fallout 3, Borderlands 2, Counterstrike and Call of Duty and third-person shooter games such as Grand Theft Auto V, Tomb Raider (2012) and Gears of War. The criterion to be considered a nonVGP was a report of little or no actionVG playing for at least the previous 12 months and no report of habitual actionVG playing during their lifetime. This resulted in twelve participants (10 males; mean age 24.0 ± 3.49 years; range 18–29 years) in the actionVGP group and twelve participants (5 males; mean age 24.7 ± 3.82 years; range 18–29 years) in the nonVGP group. The actionVGP group reported playing actionVGs for an average of 18.7 (+/- 6.7) hours per week during the last 6 months, while the nonVGP group reported playing 0 hours per week during this time.

### 2.2. Serial reaction time task

**Fig 1:**
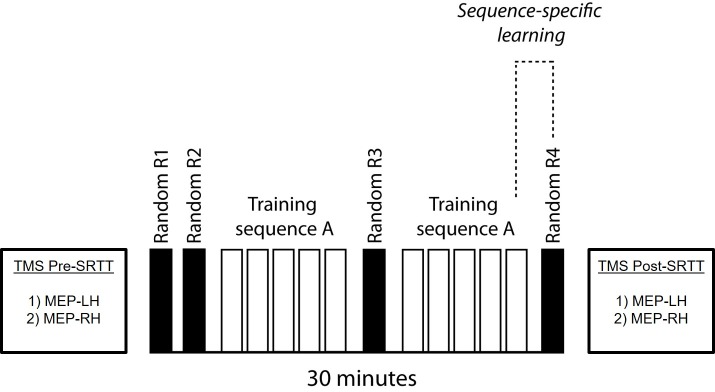
The order of the random (R) and training blocks (A) during the SRTT task is displayed. For all participants, Motor Evoked Potentials (MEP) were measured with TMS before (Pre-SRTT) and after (Post-SRTT) the SRTT over the FDI muscle representation in the left (LH) and right (RH) M1, beginning with the left hemisphere.

All participants were naive to the task. The participants were seated facing a computer screen at a distance of three feet in an upright position with the elbows flexed at a 90° angle. Both groups performed a modified version of the SRTT [22] running on Superlab (version 4.0; Cedrus, San Pedro, CA). As described in Morin-Moncet & al., (2014) [22], the task involves the presentation of three evenly positioned and horizontally aligned dots and a single asterisk that alternates between one of the four positions possible. The task lasted approximately thirty minutes. Participants were instructed to press on the key of a computer keyboard corresponding to the position of the asterisk on the computer screen as fast and accurately as possible with the correct finger (index finger for key 1, middle finger for key 2, third finger for key 3 and little finger for key 4). A correct key press was required for the following trial to appear. At each trial, the response time (RT) was calculated as the time between the presentation of the asterisk and the correct key press. Each block was composed of 10 repetitions of the same 12-item sequence for a total of 120 key presses per block. A discretionary resting period was granted between each block. Participants performed 14 blocks with their dominant, right hand (see Fig 1). The first two blocks (R1 and R2) were randomly ordered sequence blocks used to familiarize subjects with the task and to indicate initial performance, respectively. The following blocks (A1, A2, A3, A4, A5, A6, A7, A8, A9, and A10) were training blocks presenting the same 12-item repeating sequence (Sequence A: 4-2-3-1-1-3-2-1-3-4-2-4). Two additional random-sequence blocks (R3 and R4) were executed after A5 and A10, respectively, and were used to monitor the participants’ sequence learning progression. Procedural sequence-specific learning was computed as the difference in the averaged RT between the last training block A10 and the last random block R4 [32].

### 2.3. Transcranial magnetic stimulation

TMS pulses were delivered using an 8 cm figure-of-eight coil connected to a Magstim 200 (Magstim company, Whitland, Wales, UK) with a monophasic current waveform. The stimulation coil was applied flat on the scalp, held posterolaterally at a 45° angle from the mid-line to activate horizontal intracortical connections that will then activate descending corticospinal neurons [33]. A brainsight neuronavigating system (Rogue Research, Montréal, Canada) was used to ensure stable positioning throughout the experiment. The optimal stimulation sites in the left and right M1 for their respective contralateral FDI muscle activation were defined as the area eliciting MEPs of maximal amplitude at the lowest stimulation intensity. MEPs were recorded using surface electrodes positioned over the left and right FDI muscles. The electromyographic signal was amplified using a Powerlab 4 ⁄ 30 system (ADInstruments, Colorado Springs, USA), filtered with a band-pass 20–1000 Hz and digitized at a sampling rate of 4 kHz. MEPs were recorded using Scope v4.0 software (ADInstruments, Colorado Springs, USA) and stored offline for analysis. For each participant, TMS intensity used before and after the SRTT was set before the initial MEP measurement, pre-SRTT, on both hemispheres. It was adjusted at the intensity level required to elicit MEPs of ≈ 1 mV average amplitude at rest. Complete relaxation of the FDI muscle was controlled visually by monitoring the absence of EMG signal up to 100 ms before the TMS pulse. The number of MEPs excluded was marginal (i.e. less than 1%). Corticospinal excitability was measured before and after the SRTT bilaterally, beginning with the left hemisphere, by delivering ten TMS pulses to each hemisphere before and after the task, with an interstimulus interval (ISI) of 6–7 seconds. Peak-to-Peak MEP amplitudes were measured and averaged Pre- and Post- SRTT for each participant. The time between these two TMS sessions was approximately thirty minutes.

### 2.4. Data analysis

*Participants*. Age distribution and gender frequencies were compared between the actionVGP and nonVGP groups. To do so, an independent-samples T-tests with *Age* as the dependent variable and *Group* (actionVGP; nonVGP) as the between-subject factor was computed. Second, a Fisher’s exact test was conducted with *Gender* (Male; Female) as the dependent variable and *Group* (actionVGP; nonVGP) as the independent variable.

*SRTT*. Participants’ reaction times (RT) during the SRTT were filtered for aberrant data by excluding trials with RT lesser than 101 ms and greater than 1500 ms, which represented 1.8% of total trials. The presence of sequence-specific procedural learning was tested by comparing the participants’ averaged RT between the last repeating sequence block (A10) and the last random sequence block (R4) with the right hand. A mixed ANOVA with *Learning* (A10; R4) as the within-subject factor and *Group* (actionVGP; nonVGP) as the between-subject factor was computed.

*TMS*. TMS intensities defined as a percentage of the maximal device output, set before the SRTT practice for each participant’s left and right M1, were compared using a mixed ANOVA with *Hemisphere* (Left; Right) as the within-subject factor and *Group* (actionVGP; nonVGP) as the between-subject factor. Changes in corticospinal excitability originating in the left and right M1 were assessed by comparing the participants’ averaged MEP amplitudes before and after training using two separate mixed ANOVAs, one for the left M1 and a second for the right M1, with *Time* (Pre-SRTT; Post-SRTT) as the within-subject factor and *Group* (actionVGP; nonVGP) as the between-subject factor. Post-Hoc analyses were conducted accordingly with independent and paired samples T-tests using a Bonferroni correction for multiple comparisons.

## 3. Results

### 3.1. Participants

An independent-samples T-tests showed no significant difference of *Age* (*t* = -0.446; *p* = 0.66) *between* the actionVGP (M = 24.0 ± 3.49 years) and the nonVGP groups (24.7 ± 3.82 years). Likewise, the Fisher’s exact test indicated no significant difference in the frequencies of males and females between both groups (*p* = 0.089).

### 3.2. SRTT

**Fig 2:**
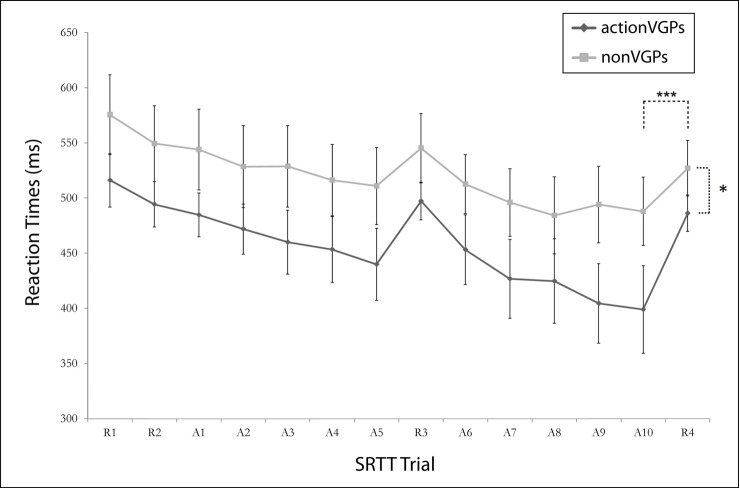
The general trend of the average reaction times (RT) are displayed in random and sequence blocks on the SRTT. However, the analysis was conducted on blocks A10 and R4. Motor sequence learning was evidenced with a significant difference in RT between A10 and R4 among both groups (*F* = 22.324; p < 0.001). In addition, the actionVGP group had faster RT on A10 and R4 compared to nonVGP (*F* = 5.874; p = 0.024) which suggest enhanced visuomotor processing abilities. *** *p*<0.001

The SRTT results can be observed in Fig 2 and Table 1. A significant effect of *Learning* (*F*_(1,22)_ = 22.324; *p* < 0.001) showed that procedural motor learning occurred for all the participants. The mixed ANOVA also revealed a significant effect of *Group* (*F*_(1,22)_ = 5.874; *p* = 0.024) which indicated that actionVGPs performed overall faster RTs compared to nonVGPs. In sum, actionVGPs displayed higher visuomotor performance in the SRTT but did not display an advantage for procedural motor learning.

**Table 1:** Summary of SRTT and TMS results.

**SRTT**	**A10 (ms)**	**R4 (ms)**	***Learning***	***Group***	***Interaction***
actionVGP	419 ± 58.88	485 ± 40.9	F_(1, 22)_ = 22.324	F_(1, 22)_ = 5.874	F_(1, 22)_ = 3.245
nonVGP	487 ± 77.22	526 ± 62.71	*p* < 0.001	*p* = 0.024	*p* = 0.085
**TMS Intensity**	**Left M1 (% max)**	**Right M1 (% max)**	***Hemisphere***	***Group***	***Interaction***
Pre-SRTT					
actionVGP	47.411 ± 8.94	47.667 ± 10.04	F_(1, 22)_ = 0.5	F_(1, 22)_ = 4.404	F_(1, 22)_ = 0.32
nonVGP	55.25 ± 12.57	55.5 ± 12.688	*p* = 0.487	*p* = 0.048	*p* = 0.577
**TMS MEP**	**Pre-SRTT (mV)**	**Post-SRTT (mV)**	***Time***	***Group***	***Interaction***
*Left M1*					
actionVGP	1.081 ± 0.185	1.725 ± 0.479	*-*	*-*	F_(1, 22)_ = 5.317
nonVGP	0.928 ± 0.218	1.147 ± 0.531			*p* = 0.031
*Right M1*					
actionVGP	1.101 ± 0.151	1.277 ± 0.48	F_(1, 22)_ = 5.605	F_(1, 22)_ = 0.004	F_(1, 22)_ = 0.8903
nonVGP	0.976 ± 0.162	1.386 ± 0.647	*p* = 0.027	*p* = 0.95	*p* = 0.355

Results of the mixed ANOVAs conducted for the SRTT and the TMS are displayed. For the SRTT, the means and standard deviations of the participants RTs on blocks A10 and R4 are expressed in milliseconds (ms). For the TMS intensity measures, the means and standard deviations represent the percentage of the stimulation device’s maximum output. For the TMS MEP, the means and standard deviations of the participants’ MEP amplitudes are expressed in millivolts (mV).

### 3.3. TMS

Results and descriptive statistics are displayed in Table 1. A mixed ANOVA comparing TMS intensities used to deliver single pulses to the participant’s left and right M1 before the SRTT indicated no significant effect of *Hemisphere* (*F*_(1,22)_ *=* 0.5; p = 0.487) and a significant effect of *Group* (*F*_(1,22)_ = 4.404; p = 0.048). Taken together, these results suggest an increased level of TMS intensity required to elicit MEP amplitudes of 1 mV on average over ten trials before the SRTT in the nonVGP compared to the actionVGP group, without difference between their left and right M1.

**Fig 3:**
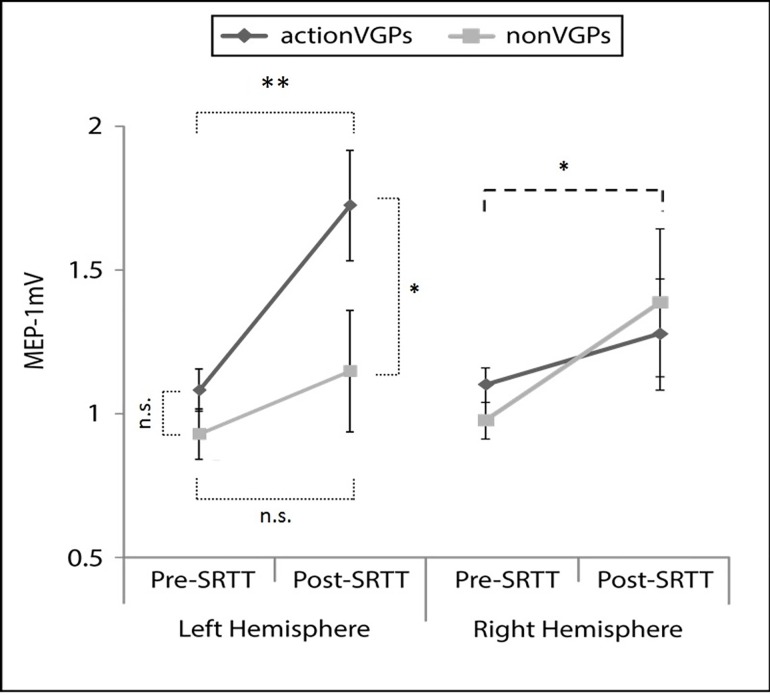
Measures of cortical excitability in the motor cortical representation of the right (Left Hemisphere) first dorsal interosseus (FDI) muscle before (Pre-) and after (Post-) SRTT among actionVGPs and nonVGPs are presented here. A significant *Time* X *Group* interaction (*F =* 5.317; p = 0.031) indicated increased MEP amplitude in the LH from Pre-SRTT to Post-SRTT for the actionVGP group (*t* = 4.681; *p* < 0.01) while the nonVGP group remained stable (*t* = 1.788; *p* = 0.404). In addition, the results showed a non-significant group difference pre-SRTT (*t*_(22)_ = 1.845; p = 0.316) and a significant difference post-SRTT (*t*_(22)_ = 2.794; p = 0.044). For the Right Hemisphere, both groups showed increased MEP amplitudes after the practice on the SRTT (F = 5.605; p = 0.027) without differences between groups (F = 0.004; p = 0.95).*p<0.05, **p<0.01

The corticospinal excitability results can be observed in Fig 3 and Table 1. For the dominant (left) hemisphere, a mixed ANOVA revealed a significant *Time* X *Group* interaction (*F*_(1,22)_ *=* 5.317; *p* = 0.031). Post-Hoc analyses using paired samples *t*-tests indicated a significant increase in corticospinal excitability (MEP amplitude) from Pre-SRTT to Post-SRTT for the actionVGP group (*t*_(11)_ = 4.681; *p* < 0.01) while MEP amplitudes remained stable before and after training for the nonVGP group (*t*_(11)_ = 1.788; *p* = 0.404). In addition, two independent-samples T-tests were performed Post-Hoc to compare left M1 MEP amplitudes between the actionVGP and the nonVGP groups at two time points, before and after SRTT practice. The results showed a non-significant group difference pre-SRTT (*t*_(22)_ = 1.845; p = 0.316) and a significant difference post-SRTT (*t*_(22)_ = 2.794; p = 0.044), with the actionVGPs displaying greater MEP amplitudes post-SRTT training compared to the nonVGPs.

For the non-dominant (right) hemisphere, a mixed ANOVA revealed a significant effect of *Time* (*F*_(1,22)_ = 5.605; *p* = 0.027) and a non-significant effect of group (*F*_(1,22)_ = 0.004; *p* = 0.95), suggesting a significant increase in corticospinal excitability following SRTT training in without differences between the actionVGP group and the nonVGP group.

## 4. Discussion

The present study provides evidence for the association between enhanced visuomotor performance in actionVGPs and increased corticospinal plasticity in M1, as measured by activity-dependent changes in MEP amplitudes and corticospinal excitability at rest. We also did not observe a relationship between video game playing experience and the ability to learn new sequences more efficiently. Instead, our results support the notion that actionVGPs display higher overall visuomotor speed, but not necessarily enhanced procedural motor learning.

Why could actionVG experience affect one motor process and not the other? Some evidence does show that procedural motor learning and general visuomotor performance are independent processes. For example, in a meta-analysis performed on seventy studies, Hardwick et al. (2013) [34] found evidence that visuomotor tasks focusing on completing novel stimulus-response patterns are differentiated from procedural learning tasks by their underlying neural activity patterns. Specifically, visuomotor tasks showed stronger activations in the basal ganglia and the cerebellum, whereas the procedural learning tasks showed increased activations in cortical structures and the thalamus. Similarly, Doyon et al. (2003) [35] found neuroimaging evidence for the differentiation between visuomotor and procedural learning tasks, with the former relating to cortico-cerebellar activity and the latter relating to cortico-striatal activity [36]. It is therefore possible that actionVG experience uniquely enhances visuomotor performance mediated by cortico-cerebellar connections but not procedural learning performance mediated by cortico-striatal connections.

What mechanisms might actionVG experience modulate to produce increased corticospinal plasticity in M1 that supports better visuomotor performance? Past research has established that practicing a visuomotor task results in the rapid increase in corticospinal excitability in M1 as part of the early stages of motor skill consolidation [37,38]. These changes in cortical plasticity are mediated by transient changes in the homeostatic balance of intracortical inhibitory mechanisms and excitatory corticospinal neural pathways that are accurately measured with TMS [39,40]. Based on this evidence, we could hypothesize that practice with actionVGs might reduce the threshold needed to increase the homeostatic balance between inhibitory and excitatory mechanisms that underlie increased corticospinal plasticity during the rapid consolidation phase of a novel visuomotor tasks. Indeed, evidence for long-term training effects on the sensorimotor system supports this notion. For example, participants who trained in a rapid tapping task in 24 sessions over a period of 4 weeks showed increases in motor speed as well as increased MEP amplitudes in the contralateral M1 over one week [41]. At a neurophysiological level, training improvements in visuomotor performance were associated with cortical reorganization and strengthening of connections in M1 that persisted over several months [25,26,41,42]. Based on this evidence, it is possible that experience with actionVGs could train M1 in a similar fashion to experimental motor training protocols [41].

Interestingly, the TMS intensity required to elicit MEP amplitudes of 1 mV at rest before the SRTT practice was decreased among actionVGPs compared to nonVGPs in the left and right M1. This discrepancy may reflect variations in corticospinal neuron sensitivity to TMS intensity in both groups, since different intensity levels were required to elicit MEPs of similar amplitudes at rest. Concurrently, the resting motor threshold, which is defined as the minimal TMS intensity required to produce MEPs of at least 50 μV five trials out of ten at rest [43], is another measure of the relation between TMS intensity and cortical excitability. RMTs were shown to have high inter-individual variability and to be fairly constant at the intra-individual level [44–46]. Part of this variance may be explained by skull-to-cortex distance [47–49] and white matter properties [50,51]. For instance, skull-to-cortex distance and the diffusion direction of the corticospinal tract were shown to account for up to 82% of the variance observed in the RMT, while the anterior-posterior corticospinal tract alone could contribute to 13% of the variance [50]. The latter finding is of particular interest considering the growing evidence for white matter changes through activity-dependent myelination that occurs during motor learning [52–53]. It could therefore be hypothesized that visuomotor skill learning associated with actionVG experience may contribute to alter white matter properties and in turn modulate corticospinal excitability.

The non-dominant (right) hemisphere showed increased corticospinal excitability pre- and post-SRTT for both groups. While the contralateral M1 is known to be particularly active during unilateral motor tasks, activity in the M1 ipsilateral to the trained hand has been frequently reported during unimanual motor training [54–58]. In fact, voluntary muscle contractions of the dominant hand were shown to modify the homotopic representation in the bilateral M1 and increases in cortical excitability in the ipsilateral M1 were evidenced during sequential thumb-finger opposition training compared to non-sequential movements [57,58]. Likewise, practice with the SRTT and sequential finger tapping tasks resulted in bilateral M1 cerebral blood-flow activations in neuroimaging studies [54–56]. The involvement of the ipsilateral M1 highlights the interaction between bilateral M1 during unilateral motor training [24,59–61]. A possible explanation for this interaction resides in the notion of interhemispheric competition, where the dominant hemisphere exerts an inhibitory action on the non-dominant hemisphere to supress superfluous activity originating from this hemisphere [28,57,58,62–64]. For example, a study by Tinazzi & Zanette (1998) [58] suggested that interhemispheric transfer of information occurs during motor learning to inhibit the opposing hemisphere when fine motor movements are required. These interhemispheric communications occur, at least in part, via transcallosal excitatory and inhibitory transfer mechanisms as well as subcortical networks [57,65–69]. Further substantiating this hypothesis, the unilateral disruption of M1 with repetitive TMS pulses enhances motor learning with the ipsilateral hand in a process named paradoxical facilitation [63–64]. At a neurophysiological level, interhemispheric inhibition (IHI) interacts with cortical plasticity mechanisms such as short intracortical inhibition and long afferent inhibition within the targeted hemisphere, depending on the parameters used to measure IHI [70]. However, the interaction between IHI and corticospinal excitability in the ipsilateral hemisphere of healthy individuals is less clear. For instance, rTMS down regulation of M1 excitability did not result in MEP amplitude or RMT changes in the contralateral M1 [71]. Likewise, the reduction of IHI from the dominant M1 to the non-dominant M1 observed following practice at the SRTT may occur in the absence of modulation of the MEP amplitudes or the RMT in the non-dominant M1 [24]. Thus, the increase in corticospinal excitability in the ipsilateral M1 reported here does not appear to be unprecedented. In accordance with the notion of hemispheric competition, the increased corticospinal excitability in the non-dominant hemisphere observed in the present study could reflect counterproductive activity from the ipsilateral M1 that does not appear to be modulated by extensive experience with actionVGs.

Some limitations in the present study do exist. First, we employed a cross sectional design and therefore true causality between actionVG experience and increased corticospinal plasticity cannot be inferred. A number of longitudinal training studies have previously found that video game playing causes the observed improvements in several cognitive domains [2,3,6,72]. It is therefore possible the relationships observed in our current experiment are also causal, but this needs to be studied further.

We also did not observe a significant increase in corticospinal excitability in the left M1 from pre-SRTT to post-SRTT in nonVGPs. This was unexpected as normal individuals usually exhibit this increase after the SRTT [22–24]. It is possible that the effect of increased corticospinal excitability at post-SRTT is present in nonVGPs, as the MEP amplitude did increase, however, the effect is not detectible at the current sample size. In addition, while research has shown that 10 single-pulse TMS delivered at a given intensity level over a specific muscle representation in the contralateral M1 is sufficient to provide reliable intra-session measurements of cortical excitability [73,74], recent studies suggests that 20 MEPs may be necessary to produce a stable response [75]. Thus, it is possible that the number of TMS trials compared before and after SRTT in the current study reduces the statistical power required to reveal this effect in our nonVGP group. This nonetheless does not limit our interpretations pertaining to corticospinal excitability in actionVGPs as this group did display a relative increase in MEP amplitude compared to nonVGPs at the current sample size.

In summary, our results demonstrate that underlying improvements in behavioural performance in actionVGPs are reflected in increases in corticospinal plasticity in M1. These results support the hypothesis that actionVG playing is associated with neuroplastic changes that underlie increased visuomotor processing [76]. Further studies are needed to establish the causality of this observed effect employing a longitudinal design.

## Supporting Information

S1 Dataset (XLS)
